# GelMA-glycol chitosan hydrogels for cartilage regeneration: The role of uniaxial mechanical stimulation in enhancing mechanical, adhesive, and biochemical properties

**DOI:** 10.1063/5.0160472

**Published:** 2023-09-08

**Authors:** Sattwikesh Paul, Karsten Schrobback, Phong Anh Tran, Christoph Meinert, Jordan William Davern, Angus Weekes, Udhaya Nedunchezhiyan, Travis Jacob Klein

**Affiliations:** 1 Centre for Biomedical Technologies, Queensland University of Technology (QUT), 60 Musk Ave., Kelvin Grove, Brisbane, QLD 4059, Australia; 2 School of Mechanical, Medical and Process Engineering, Faculty of Engineering, Queensland University of Technology (QUT), 2 George Street, Brisbane, QLD 4000, Australia; 3 Department of Surgery and Radiology, Faculty of Veterinary Medicine and Animal Science (FVMAS), Bangabandhu Sheikh Mujibur Rahman Agricultural University (BSMRAU), BSMRAU Road, Gazipur 1706, Bangladesh; 4 School of Biomedical Sciences, Centre for Genomics and Personalised Health, Translational Research Institute, Queensland University of Technology (QUT), 37 Kent Street, Woolloongabba, QLD 4102, Australia; 5 Gelomics Pty Ltd., Brisbane, QLD 4059, Australia; 6 ARC Training Centre for Cell and Tissue Engineering Technologies, Queensland University of Technology (QUT), Brisbane, QLD 4059, Australia

## Abstract

Untreated osteochondral defects are a leading cause of osteoarthritis, a condition that places a heavy burden on both patients and orthopedic surgeons. Although tissue engineering has shown promise for creating mechanically similar cartilage-like constructs, their integration with cartilage remains elusive. Therefore, a formulation of biodegradable, biocompatible biomaterial with sufficient mechanical and adhesive properties for cartilage repair is required. To accomplish this, we prepared biocompatible, photo-curable, mechanically robust, and highly adhesive GelMA-glycol chitosan (GelMA-GC) hydrogels. GelMA-GC hydrogels had a modulus of 283 kPa and provided a biocompatible environment (>70% viability of embedded chondrocytes) in long-term culture within a bovine cartilage ring. The adhesive strength of bovine chondrocyte-laden GelMA-GC hydrogel to bovine cartilage increased from 38 to 52 kPa over four weeks of culture. Moreover, intermittent uniaxial mechanical stimulation enhanced the adhesive strength to ∼60 kPa, indicating that the cartilage-hydrogel integration could remain secure and functional under dynamic loading conditions. Furthermore, gene expression data and immunofluorescence staining revealed the capacity of chondrocytes in GelMA-GC hydrogel to synthesize chondrogenic markers (COL2A1 and ACAN), suggesting the potential for tissue regeneration. The promising *in vitro* results of this work motivate further exploration of the potential of photo-curable GelMA-GC bioadhesive hydrogels for cartilage repair and regeneration.

## INTRODUCTION

Poor self-healing mechanisms and loading on incongruent joints may accelerate cartilage deterioration and initiate the course of osteoarthritis (OA),^1,2^ making articular cartilage regeneration challenging. Tissue engineering of articular cartilage has been investigated^3^ as an alternative to surgical procedures used for cartilage damage repair and regeneration, which frequently fail to fully restore tissue function.^4,5^ Many different cell types and scaffolding materials have been explored *in vitro*^6^ to generate neo-tissue constructs with native-like properties, with varying degrees of success. Hydrogels, three-dimensional cell culture materials that can be chemically and mechanically tuned, have shown considerable potential for cartilage tissue engineering.^7^ To enhance the formation of hydrogel-based tissue-engineered cartilage, design techniques have incorporated stimuli resembling the natural cellular environment. The fundamental biological problems of three-dimensional cartilage regeneration are producing a repair tissue with similar structural and mechanical properties to the articular cartilage^8,9^ and achieving strong integration between the host and repair tissue.^8,10–12^ Moreover, the biomechanical environment is a critical regulator of tissue growth, maintenance, and regeneration in native articular cartilage.^13^ Articular cartilage is subjected to various mechanical forces, including hydrostatic pressure, compression, shear and tension, and interstitial fluid flow-mediated frictional drag during loading.^14^ Chondrocytes experience these mechanical stimuli and regulate gene transcription, matrix biosynthesis, and turnover through mechano-transduction pathways.^15^ Depending on the nature of the load, the mechano-transduction of these signals into a biochemical response by the cells might result in increased synthesis of extracellular matrix (ECM) molecules or a pathologic response.^16^ Chondrocyte metabolism is affected by both the magnitude and the type of mechanical load applied.^17^ Furthermore, the type of mechanical load applied also appears to influence implant integration with host cartilage.^18–20^

Mechanical stimulation bioreactors, which mimic the biomechanical environment of native articular cartilage by providing compression,^21,22^ shear,^23^ hydrostatic pressurization,^24–26^ and fluid flow,^27–29^ are used to evaluate the impact of mechanical stimuli on neo-tissue formation. Moreover, the application of these stresses within a physiological range enhanced the biosynthetic response of chondrocyte-laden hydrogel for tissue-engineered articular cartilage formation and maturation.^30,31^ Mechanical stimulation, on the other hand, is a significant pathogenic component when loading is excessive and may contribute to developing degenerative disorders such as OA.^4,32,33^ It was observed that static compression suppresses matrix synthesis while dynamic loading favors chondrocyte biosynthesis.^34^ Additionally, chondrocytes are sensitive to loading frequency,^35^ the amount of force or strain applied,^35^ static pre-culture, and loading durations.^22^ The pericellular matrix is crucial for chondrocyte mechano-sensitivity^36,37^ and protects chondrocytes from significant local tissue strains,^38^ hence preventing structural damage.^39^ During compression, it was observed that collagen fibrils in the pericellular matrix played a substantial role in preserving the width and volume of the enclosed chondrocytes.^40^ Therefore, a period of free-swelling preculture is necessary to develop a pericellular matrix in a cell-laden construct prior to applying mechanical stimulation during culture.^22^

When engineered tissue is used to fill a cartilage defect, mismatches in mechanical properties will result in different stresses in the engineered and native tissue during joint loading. This can affect both the loading-induced cellular stimulation and the integration between the engineered and native tissues. The gene expression of neighboring chondrocytes has been demonstrated to be affected by abnormal load distribution at the margin of a focal cartilage defect.^41^ Finite element modeling of a cartilage defect filled with a tissue-engineered construct revealed a difference in mechanical characteristics (with constructs weaker than cartilage) that generated stress concentrations in the native cartilage near the defect. Although mechanical stimulation is crucial in regulating matrix turnover in healthy cartilage, without robust integration between repair and native tissue, loading may generate micromotion and hinder matrix synthesis at the border.^42^ Therefore, the heterogeneous variable load distribution across the implant-to-native cartilage interface may affect mechano-transduction pathways and chondrocyte biosynthesis and, at extreme levels, induce cell death.

We have been developing visible-light cross-linked GelMA-glycol chitosan (GelMA-GC) hydrogels with promising mechanical and adhesive properties. However, it is unclear how encapsulated chondrocytes will respond to physiological mechanical stimuli in this hydrogel system, and if the hydrogel and cartilage will remain well-integrated following such mechanical loading. We hypothesized that chondrocytes would benefit from intermittent uniaxial loading regimes appropriate for tissue growth in a cartilage defect model. Moreover, we also hypothesized that mechanical stimulation would influence cartilage-hydrogel integration in a physiologically similar environment. To test those hypotheses, we encapsulated bovine chondrocytes in Ru/SPS cross-linked GelMA-GC hydrogel within a bovine cartilage ring and subjected them to 14 days of *in vitro* uniaxial mechanical stimulation, and evaluated the construct's mechanical and adhesive properties, as well as cell viability and production of extracellular matrix.

## RESULTS

### Mechanical test: Micro-indentation

**FIG. 1.**
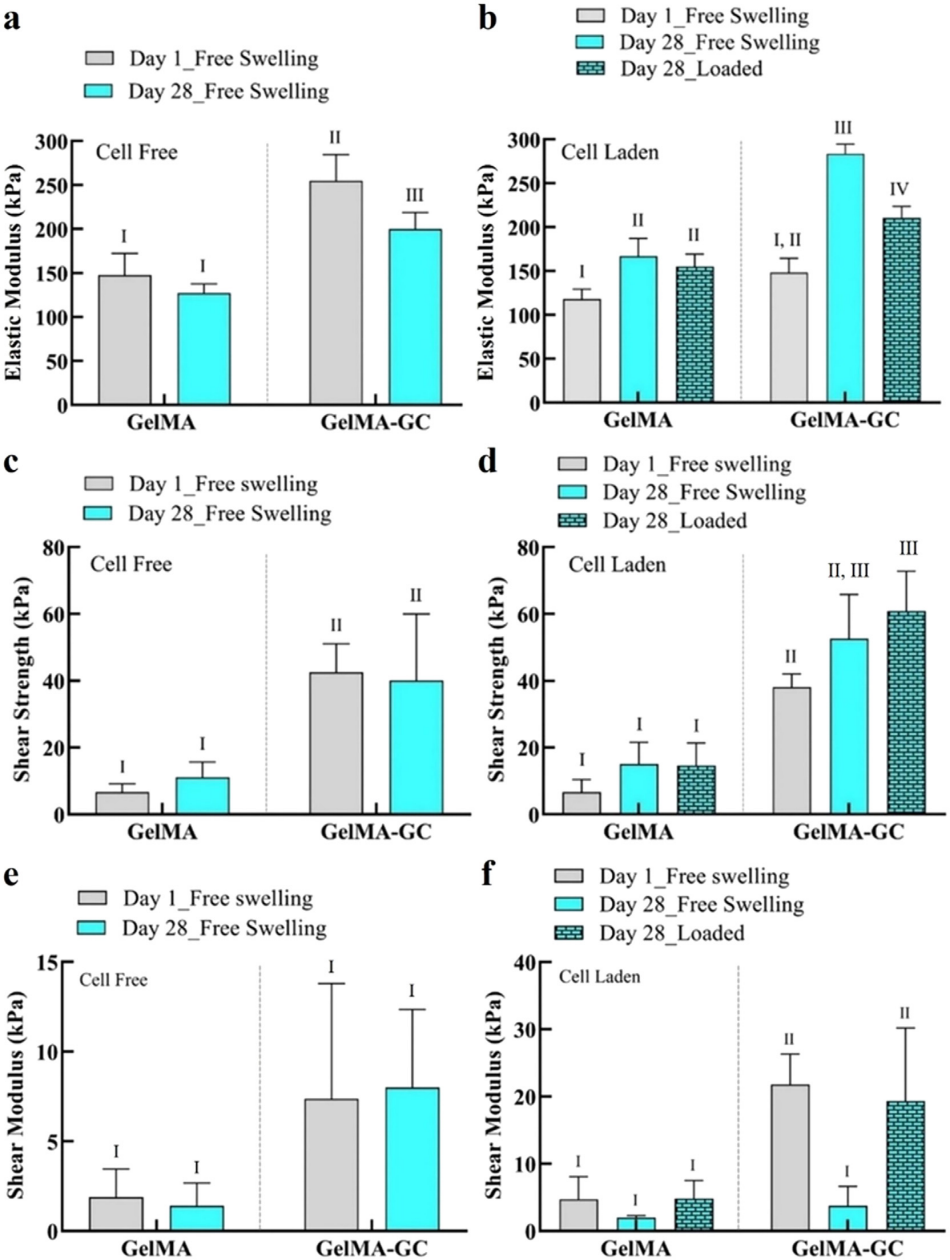
Effects of glycol chitosan and uniaxial mechanical stimulation on the mechanical and adhesive properties of hydrogels. Cell-free, cell-laden, free-swelling, and loaded GelMA (15%; w/v) and GelMA-GC (GelMA 15% and GC 1%, w/v) hydrogels cross-linked with Ru/SPS photo-initiators at 405 nm into the central defect of bovine articular cartilage on day 1 and 28. Elastic modulus calculated from the micro-indentation test by cellscale micro-tester at 37 °C in PBS (pH = 7.4) bath (a) cell-free and (b) cell-laden hydrogels. Shear strength of (c) cell-free and (d) cell-laden hydrogels measured after push-out test using an Instron 5567 tester with a nonporous indenter and a 5 N load cell at 37 °C in PBS (pH = 7.4) bath. Shear modulus of (e) cell-free and (f) cell-laden hydrogels measured after push-out test using an Instron 5567 tester with a nonporous indenter and a 5 N load cell at 37 °C in PBS (pH = 7.4) bath. Groups that do not share a common Roman numeral are statistically different (p < 0.05). GelMA (15%, w/v) hydrogels were considered as control. Sample size n = 4 per group; error bars: Mean + SD.

The elastic modulus (E) of GelMA and GelMA-GC hydrogels was calculated using the Hertz contact model for a spherical indenter.^43^ GC significantly enhanced the E of cell-free hydrogels compared to GelMA hydrogels on day 1. On day 28, GelMA-GC hydrogels also showed a significantly higher E (199.8 ± 19.1 kPa) compared to GelMA hydrogels (127.0 ± 10.7 kPa). Cell-free GelMA-GC hydrogels showed a decreasing trend of E as culture continued [Fig. 1(a)]; however, an opposite trend was observed when cells were included [Fig. 1(b)].

The E of all cell-laden groups was increased after 28 days of culture, with GelMA hydrogels increasing 1.4-fold (increased from 118.1 ± 11.3 to 166.8 ± 20.2 kPa) and GelMA-GC hydrogels increasing 1.9-fold (148.3 ± 16.4 to 283.7 ± 10.9 kPa) compared to day 1. Moreover, in the loaded conditions, GelMA and GelMA-GC hydrogels showed around a 1.3-fold (increased from 118.1 ± 11.3 to 155.2 ± 14.0 kPa) and a 1.4-fold increase (from 148.3 ± 16.4 to 210.5 ± 13.2 kPa) of modulus compared to their day 1 free-swelling controls. Cell-laden free-swelling GelMA-GC hydrogels showed the highest E (283.7 ± 10.9 kPa) on day 28 of culture [Fig. 1(b)]. The mechanical properties of GelMA hydrogels, however, were unaffected by loading on day 28 of culture as compared to their respective free-swelling groups [Fig. 1(b)]. However, loaded GelMA-GC hydrogels had significantly higher E than loaded GelMA hydrogels [Fig. 1(b)].

### Adhesion test: Push-out

The adhesive characteristics of cell-free, cell-laden, free-swelling, and loaded GelMA and GelMA-GC hydrogels were evaluated using a push-out test^44,45^ [Fig. 7(d)]. GC considerably improved the adhesive strength of hydrogels cultured in all conditions [Figs. 1(c) and 1(d)]. Cell-free GelMA-GC hydrogels showed significantly higher shear strengths (42.6 ± 8.5 kPa) and (40.1 ± 19.8 kPa) compared to GelMA hydrogels (6.7 ± 2.5 kPa) and (11.1 ± 4.7 kPa) on day 1 and 28, respectively [Fig. 1(c)]. Moreover, both cell-laden free-swelling and loaded GelMA-GC hydrogels had significantly higher shear strengths compared to their respective GelMA controls [Fig. 1(d)]. Loaded GelMA-GC hydrogels showed the highest shear strength (60.8 ± 12.0 kPa) after 28 days culture [Fig. 1(d)]. Moreover, loading substantially increased the shear strength from 38.1 ± 3.9 kPa on day 1 to 60.8 ± 12.0 kPa on day 28. Although the values were not statistically different, the loading resulted in a 1.2-fold increase in shear strength of GelMA-GC hydrogels (increased from 52.6 ± 13.3 to 60.8 ± 12.0 kPa) as compared to cell-laden day 28 of free-swelled GelMA-GC hydrogels [Fig. 1(d)].

The shear moduli of cell-free GelMA-GC and GelMA hydrogels were statistically similar and remained unchanged over 4 weeks of culture [Fig. 1(e)]. In contrast, cell-laden GelMA-GC hydrogels had a higher shear modulus than GelMA controls on day 1 [Fig. 1(f)]. Moreover, loaded GelMA-GC hydrogels showed significantly higher shear modulus compared to free-swelling GelMA and GelMA-GC hydrogels on day 28 [Fig. 1(f)]. Throughout the culture period, GelMA hydrogels showed lower shear modulus compared to GelMA-GC hydrogels cultured in both cell-free and cell-laden conditions, except at day 28 free-swelling. Loading did not influence the shear modulus of GelMA hydrogels [Fig. 1(f)].

### Viability and cell proliferation

**FIG. 2.**
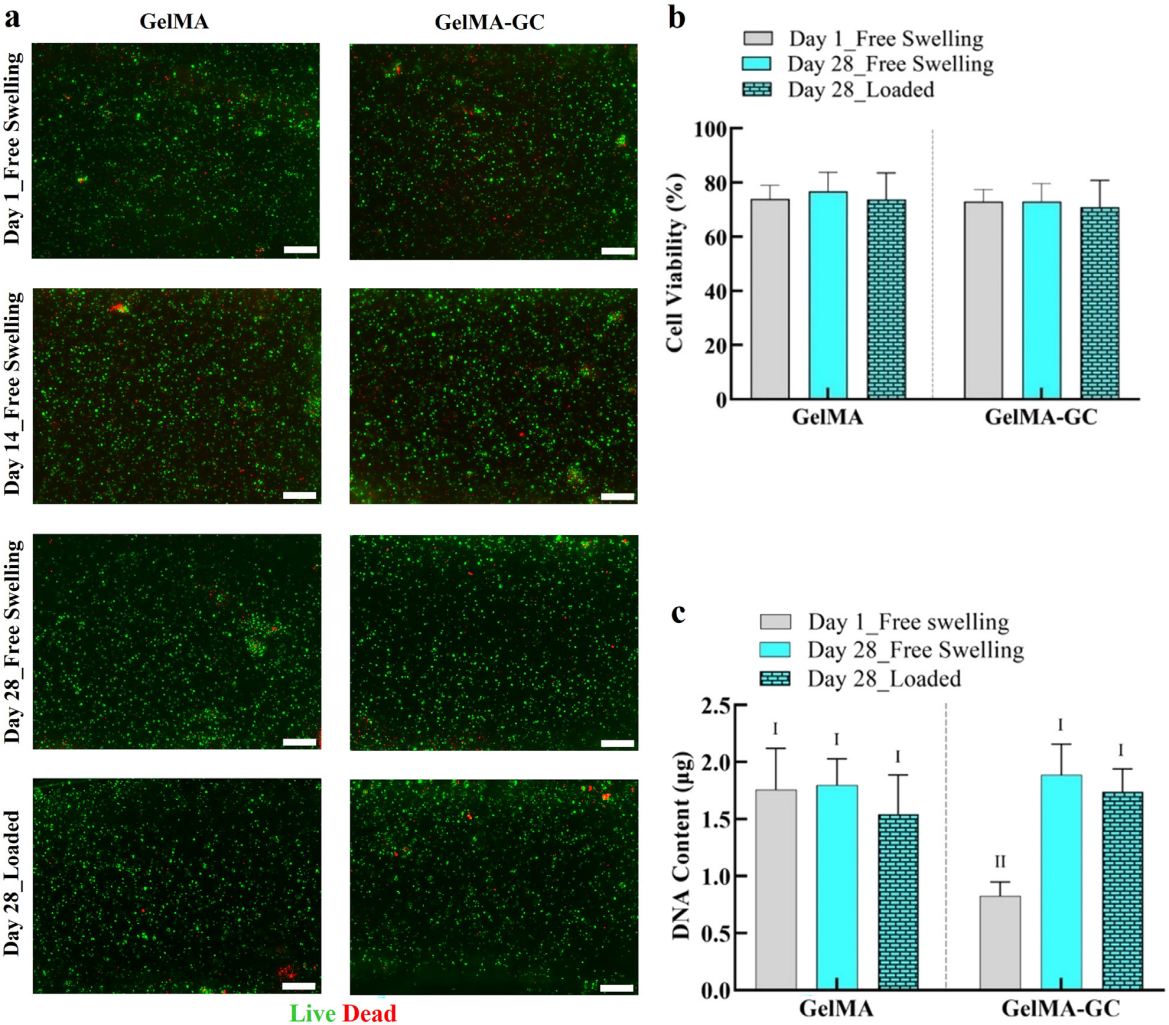
Cell viability and proliferation of bovine articular chondrocytes in GelMA and GelMA-GC hydrogels. Cell viability of bovine articular chondrocytes in cell-laden free-swelled and loaded GelMA (15%, w/v) and GelMA-GC (GelMA 15% and GC 1%, w/v) hydrogels cross-linked by Ru/SPS photo-initiators at 405 nm on day 1 and 28 of culture. (a) Representative live/dead images of chondrocytes encapsulated (seeding density 8.3 × 10^6^ cells per ml of hydrogel) in GelMA and GelMA-GC hydrogels. Living cells appear green, dead cells appear red. Scale bars: 200 *μ*m. (b) Cell viability represented as a percentage of the total number of cells that were alive. (c) Total DNA content of hydrogel constructs. Groups that do not share a common Roman numeral are statistically different (p < 0.05). GelMA (15%, w/v) hydrogels were considered as control. Sample size n = 4 per group; error bars: Mean + SD.

To determine the cytocompatibility of GelMA and GelMA-GC hydrogels, a cell viability study was conducted on days 1 and 28 of culture. The cell vitality of GelMA-GC hydrogels was high (>70%) in both free-swelling and loaded groups and comparable to GelMA hydrogels on days 1 and 28 [Figs. 2(a) and 2(b), supplementary material Fig. S3(c)].

To visualize the impact of intermittent uniaxial loading on hydrogels, cross-sectional images of hydrogels following a live/dead assay were photographed (supplementary material Fig. S1). After 14 days of loading (day 28 of culture), a thin layer of dead cells was identified on the bottom surface of the GelMA-GC hydrogels [supplementary material Fig. S1(d)]. However, overall hydrogel cross sections revealed comparable cell viability in all groups following the cessation of the loading period on day 28 of culture [Figs. 2(a) and 2(b)].

To comprehensively examine the integration and cell viability of distinct cartilage-hydrogel areas, we performed live/dead staining of the whole cartilage-hydrogel construct (supplementary material Fig. S2). GelMA-GC hydrogels demonstrated robust cartilage-hydrogel integration after 4 weeks of culture, but GelMA hydrogel failed to maintain a strong cartilage-hydrogel integration (supplementary material Fig. S2). Moreover, we observed a substantial number of live cells in the specific areas of cartilage-hydrogel constructs of both hydrogels [supplementary material Figs. S2(a)–S2(d)].

### Biochemical analysis

**FIG. 3.**
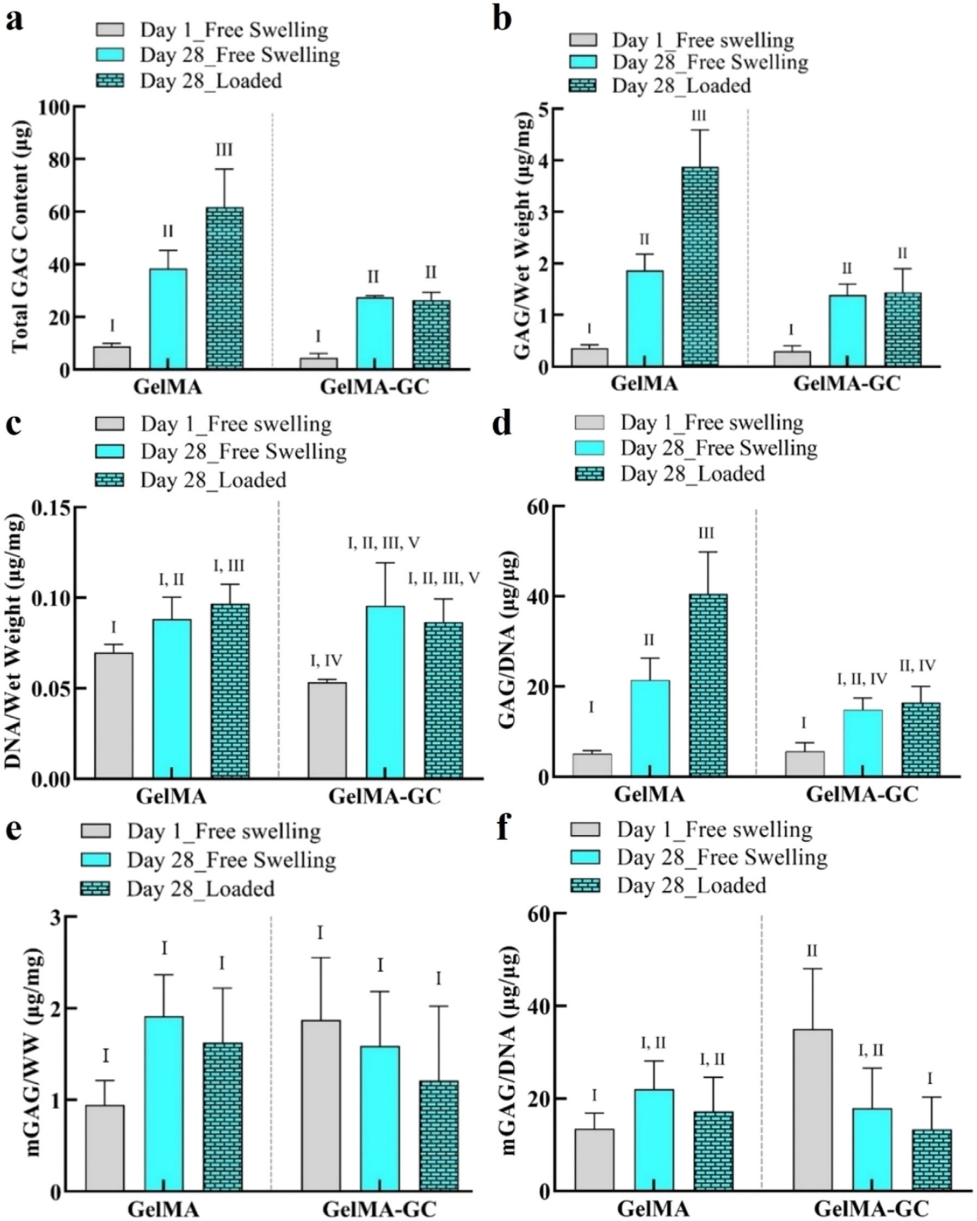
Biochemical properties of cell-laden GelMA and GelMA-GC hydrogels. Biochemical properties of cell-laden free-swelled and loaded GelMA (15%, w/v) and GelMA-GC (GelMA 15% and GC 1%, w/v) hydrogels cross-linked by Ru/SPS photo-initiators at 405 nm on day 1 and 28 of culture. Bovine articular chondrocytes were encapsulated (seeding density 8.3 × 10^6^ cells per mL of hydrogel) in GelMA and GelMA-GC hydrogels and cultured at 37 °C with 5% CO_2_ for 28 days with media changes twice in a week. (a) Total GAG content and (b) total GAG content normalized to hydrogel wet weight. (c) DNA content normalized to hydrogel wet weight and (d) total GAG content normalized to DNA content. GAG secreted into the media on day 1 and 28 of culture (e) normalized to hydrogel wet weight and (f) DNA content. Groups that do not share a common Roman numeral are statistically different (p < 0.05). GelMA (15%, w/v) hydrogels were considered as control. Sample size n = 4 per group; error bars: Mean ± SD.

We determined the GAG and DNA content of hydrogels on day 1 and 28 by DMMB and Picogreen assays, respectively, immediately after the push-out test. A significant accumulation of GAG in the hydrogels of each group was observed on day 28 in both groups compared to day 1 of culture [Fig. 3(a)]. Loading significantly increased GAG accumulation in GelMA hydrogels compared to free-swelling day 1 and 28 GelMA hydrogels. The effect of loading on GAG accumulation was also significant in GelMA-GC hydrogels relative to their day 1 free-swelling hydrogel; however, it was comparable to their day 28 free-swelling hydrogels [Fig. 3(a)]. When GAG content was normalized to the wet weight of hydrogels, similar trends were observed in both groups [Fig. 3(b), supplementary material Fig. S3(b)].

Total DNA content was significantly increased in GelMA-GC hydrogels on day 28 compared to their day 1 free-swelling hydrogel. However, GelMA hydrogels maintained similar DNA content irrespective of days of culture studied. Moreover, GelMA-GC hydrogels showed comparative DNA content to their GelMA hydrogel control on day 28 of the culture [Fig. 2(c)]. Intermittent uniaxial loading had no influence on the DNA content of both hydrogels on day 28 in their respective group [Fig. 2(c)]. A similar phenomenon was observed when DNA content was normalized with the wet weight of hydrogels at day 28 of culture [Fig. 3(c)]. When retained GAG was normalized to DNA content, both groups showed a consistently increasing trend from day 1 to day 28 of the culture [Fig. 3(d)]. We also measured the secreted GAG in the media (mGAG) to investigate the ability of hydrogel matrices to retain newly synthesized GAG. On day 28 of culture, GelMA and GelMA-GC hydrogels showed comparable GAG secretion in the media normalized to wet weight where loading did not affect GAG secretion [Fig. 3(e)]. When mGAG was normalized to DNA content, GelMA-GC hydrogels showed significantly higher value compared to GelMA-only hydrogels on day 1. However, on day 28 of culture, both free-swelling and loaded GelMA-GC hydrogels and GelMA hydrogels demonstrated comparative values [Fig. 3(f)]. In addition, the total amount of GAG secreted into the media was determined at each media change in both cell-free and cell-laden cartilage-hydrogel construct groups and presented the data schematically [supplementary material Fig. S3(a)].

### Immunofluorescence analysis

**FIG. 4.**
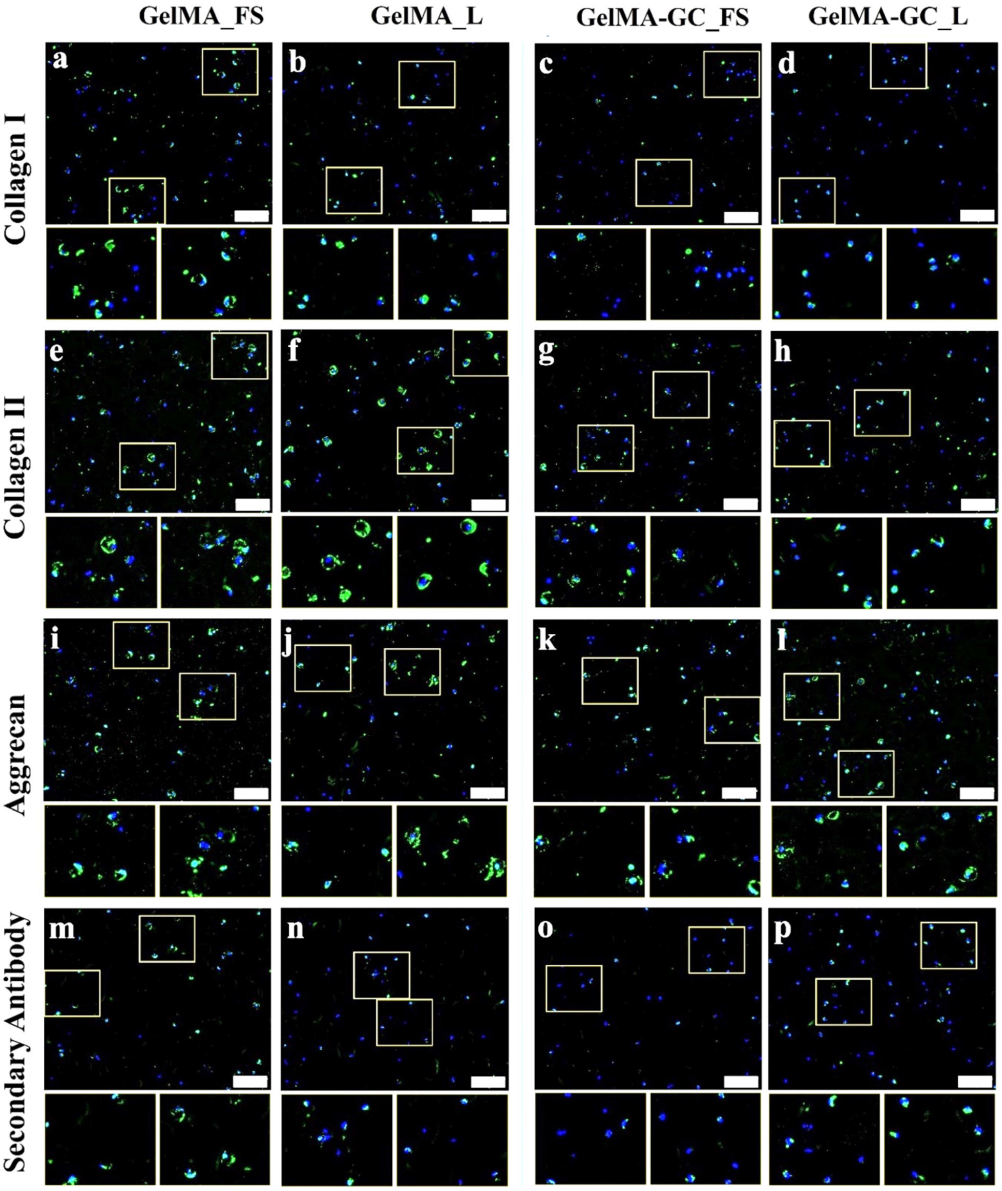
Extracellular matrix in free-swelling (FS) and uniaxially loaded (L) constructs after 28 days of culture. Immunofluorescence staining for (a)–(d) collagen I, (e)–(h) collagen II, (i)–(l) aggrecan, and (m)–(p) secondary antibody of statically cultured and loaded GelMA (15 %, w/v) and GelMA-GC (GelMA 15% and GC 1%, w/v) hydrogels cross-linked by Ru/SPS for 5 min at 405 nm LED light. Immunoreactive regions for collagen I, collagen II, and aggrecan appear green. Secondary antibody as a negative control appear green. Nuclei were counterstained with DAPI (blue). Scale bar: 200 *μ*m. White boxes on main images are shown at higher magnification directly below the main image.

Immunofluorescent images of GelMA and GelMA-GC hydrogels were used to evaluate the synthesis and accumulation of collagen I, II, and aggrecan markers in encapsulated chondrocytes. Slides stained with only secondary antibody were utilized as negative controls. Collagen I immunoreactivity was observed in all free swelling and loaded GelMA hydrogels, while the loaded group had reduced immunoreactivity [Figs. 4(a) and 4(b), supplementary material Fig. S4(a)]. Although collagen I immunoreactivity was observed in day 1 free swelling GelMA-GC hydrogels [supplementary material Fig. S4(e)], this was significantly diminished in both the swelling and loaded groups after 28 days of culture [Figs. 4(c) and 4(d)]. The collagen II immunoreactivity was shown to be higher in the free-swelled and loaded GelMA groups than in the GelMA-GC groups, regardless of days and culture conditions [Figs. 4(e)–4(h), supplementary material Figs. S4(b) and S4(f)]. All hydrogels regardless of loading condition demonstrated positive immunoreactivity to aggrecan throughout the culture period [Figs. 4(i)–4(l), supplementary material Figs. S4(c) and S4(g)]. The pattern of fluorescence in the secondary antibody controls was consistent throughout all days and conditions studied with both groups of hydrogels [Figs. 4(m)–4(p), supplementary material Figs. S4(d) and S4(h)].

### Gene expression analysis

**FIG. 5.**
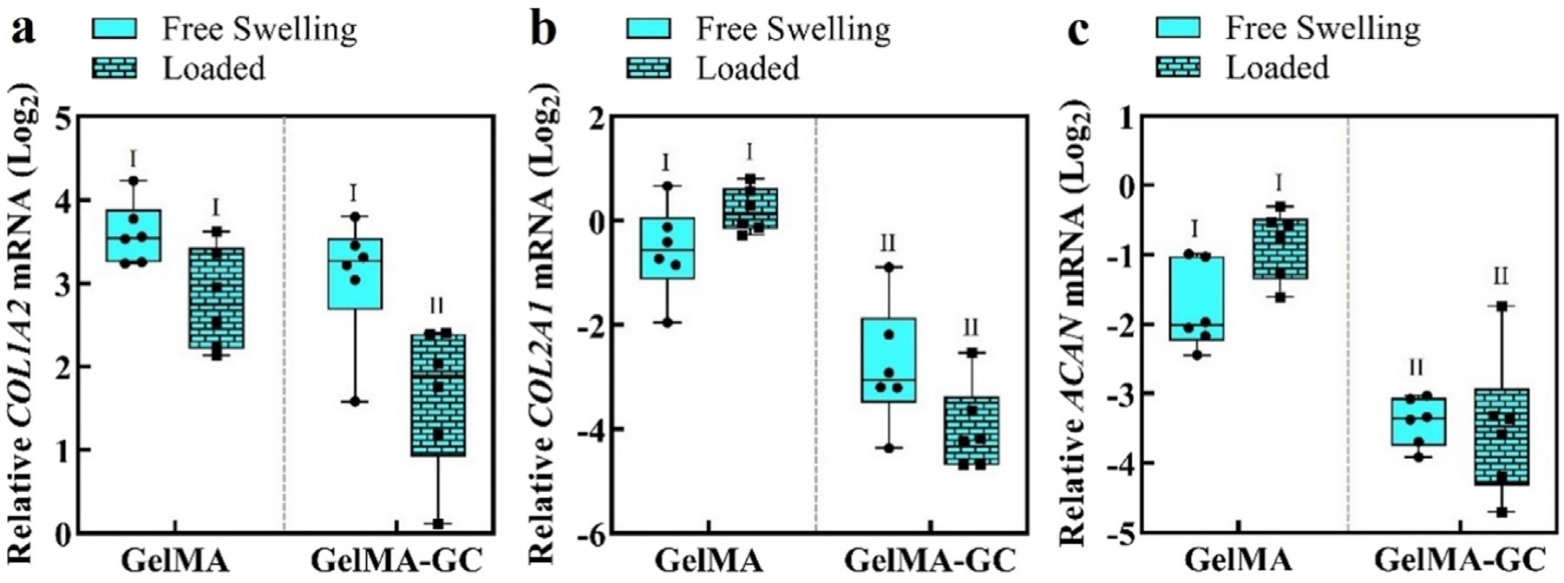
Gene expression of bovine articular chondrocytes in GelMA and GelMA-GC hydrogel constructs at 28 days of culture. Relative mRNA expression levels of (a) collagen I (COL1A2), (b) collagen II (COL2A1), and (c) aggrecan (ACAN) in statically cultured (free swelling) and intermittently loaded (loaded after 14 days pre-culture; total 28 days) GelMA (15%, w/v) and GelMA-GC (GelMA 15% and GC 1%, w/v) hydrogels cross-linked with Ru/SPS at 405 nm LED light. Gene expression is relative to the geometric mean of housekeeping genes RPL13A and B2M and presented on a log_2_ scale. Mechanically stimulated constructs were terminated 2 h after the last loading cycle had finished. Hydrogel types sharing the same Roman numeral are statistically similar, while hydrogel types with different numerals are significantly different (p < 0.05; n = 6).

To determine the effects of GC and intermittent uniaxial loading following 14 days of pre-culture conditions on gene expression, we conducted qRT-PCR at the end of the culture period. Samples from the loaded group were collected 2 h after the termination of the final loading cycle.^46^ The expression of COL1A2, COL2A1, and ACAN genes was compared to the geometric mean of the housekeeping genes RPL13A and B2M. The gene expression data demonstrated that the presence of GC lowered the chondrogenic markers COL2A1 and ACAN in comparison to GelMA-only hydrogels in both culture conditions [Figs. 5(b) and 5(c)]. Loading decreased the expression of the fibro-cartilage marker COL1A2 in GelMA-GC hydrogels, but not in the GelMA-only group [Fig. 5(a)]. There were no differences in transcript levels of the chondrogenic markers COL2A1 and ACAN between free-swelling and loaded hydrogels in any of the groups [Figs. 5(b) and 5(c)].

## DISCUSSION

To successfully repair, and ultimately regenerate, defects in articular cartilage, tissue-engineered cartilage must have mechanical properties that are compatible with the joint environment, integrate with the surrounding tissue, and support chondrocytes. This study describes *in vitro* evaluation of a natural polymer-based, photo-curable, injectable, mechanically robust, highly adhesive GelMA-GC hydrogel for cartilage defects repair and regeneration in a dynamic mechanical environment.

The mechanical properties of GelMA-GC hydrogels [∼150–250 kPa, Fig. 1(b)] were substantially higher than many hydrogels investigated for cartilage tissue engineering.^47,48^ Incorporation of GC significantly increased the E of GelMA hydrogels [Figs. 1(a) and 1(b)], possibly due to the formation of H-bonds between GC and the GelMA network.^49,50^ In addition, we hypothesized that the diverse cross-linking mechanisms of Ru/SPS, acting through radical polymerization and di-tyrosine bonding with available tyrosines, also contributed to the improvement of the E of GelMA-GC hydrogels.^51,52^ We further hypothesize that, as a polycationic polymer, GC might have formed ionic interactions with the sulfate anions, such as those in chondroitin sulfate stemming from the surrounding cartilage or the encapsulated cells, potentially further contributing to the E. However, additional study is required to confirm this hypothesis. In 28-day cultures, both cell-laden hydrogels showed an increase in resistance to compression over time of culture as part of the successful chondrogenic differentiation process [Fig. 1(b)]. When chondrocytes were encapsulated in this hydrogel and cultured free-swelling for 28 days demonstrated a higher E (283.7 ± 10.9 kPa). Furthermore, when these constructs were loaded for 14 days, the E (210.5 ± 13.2 kPa) was still encouraging [Fig. 1(b)]. In articular cartilage, GAG significantly contributes to the compressive characteristics by attracting water, which causes swelling restricted by the cross-linked collagen network, increasing hydrostatic pressure and compressive stiffness of the tissue.^53^ After 28 days of culture, GelMA-GC hydrogels exhibited a higher E than GelMA hydrogels despite a similar amount of GAG accumulation [Figs. 1(b) and 3(a)], indicating that both GAG accumulation and molecular interactions played an important role in increasing the stiffness of GelMA-GC hydrogels. Moreover, on day 28 of culture, the E of free-swelling and loaded GelMA-GC hydrogels containing cells increased sharply compared to day 1 [Fig. 1(b)], suggesting the ability to maintain their structural integrity and mechanical properties over time. Contrary to our expectations, on 28 days of culture in the same group, loading did not increase the modulus of GelMA-GC hydrogels compared to their free-swelling controls [Fig. 1(b)]. This may be due to the construct-retained GAG level, which was unaffected by loading in that hydrogel [Fig. 3(a)]. While the E of GelMA-GC hydrogel is lower than that of human knee articular cartilage (∼0.5–0.7 MPa^54^), it is higher than that of visually intact superficial regions of osteoarthritic human articular cartilage (20 ± 3 kPa)^55^ as well as chitosan-glycerophosphate hydrogels (1.5–18 kPa)^56^ that is the basis of clinically applied BST-Cargel. Based on the increase in E of cell-laden GelMA-GC hydrogels from day 1 to day 28 of *in vitro* culture, we hypothesize that the E would likely further increase after *in vivo* implantation, as it is expected that implanted cells will generate more new cartilage tissue as the hydrogel degrades.

**FIG. 6.**
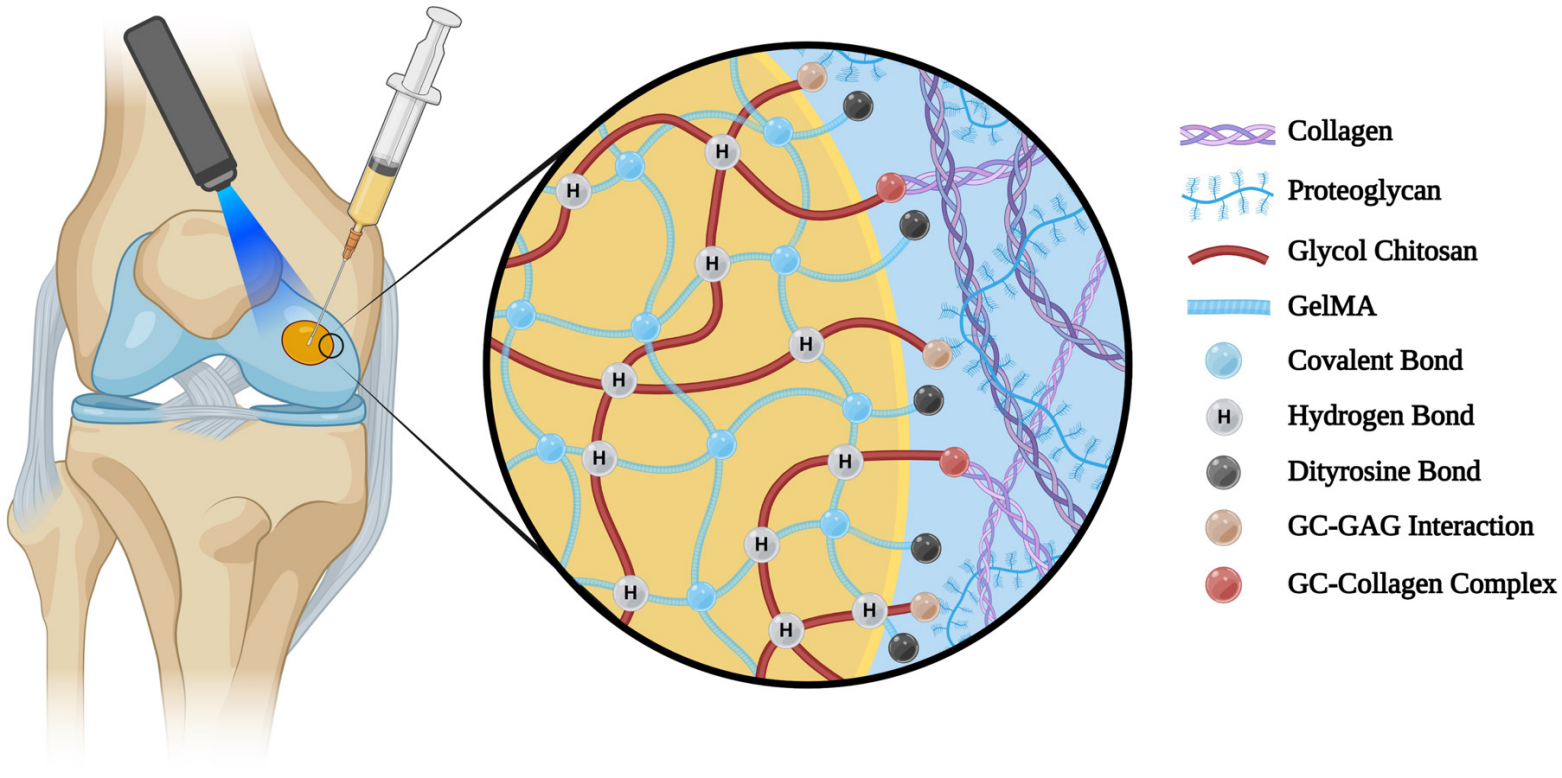
Schematic illustration of adhesion mechanisms of Ru/SPS cross-linked GelMA-GC hydrogels with native cartilage. Figure created with BioRender.com.

Improving the integration of the grafted material and cartilage tissue is essential to ensure the long-term success of the implantation. Suboptimal integration may lead to graft instability and failure.^57^ The adhesive properties of our hydrogel system were determined based on the shear strength data recorded from the push-out test. Although the shear strengths of cell-free and cell-laden GelMA-GC hydrogels on day 1 were comparable (38–42 kPa) [Figs. 1(c) and 1(d)], this is higher than other GelMA hydrogels and previously reported bioadhesive hydrogels.^44,45,58–60^ Furthermore, after a 28-day study period, GelMA-GC hydrogels had significantly higher shear strength than GelMA hydrogels across all conditions, with the highest shear strength recorded in loaded GelMA-GC hydrogels (60.8 ± 11.9 kPa) [Fig. 1(d)]. This may be due to the synergistic effects of ionic interactions between polycationic GC and biological surfaces containing anionic GAGs^61–64^ and collagens,^65,66^ Ru/SPS-mediated di-tyrosine bonding between GelMA and proteins^59^ at the defect site, or other mechanisms (Fig. 6). Mechanical stimulation also resulted in a considerable rise in shear modulus of GelMA-GC hydrogel compared to free-swelled hydrogels on day 28 [Fig. 1(f)], indicating that it can provide greater physical support for the cartilage, reduce wear and tear, and maintain structural integrity during loading. On the contrary, the effect of mechanical stimulation on enhancing the shear modulus of GelMA hydrogels was negligible [Fig. 1(f)]. The full z-stack images of cartilage-hydrogel constructs cultured in both free-swelling and loaded conditions revealed promising integration of GelMA-GC hydrogels with native cartilage [supplementary material Figs. S2(b) and S2(d); yellow arrows]. However, a notable gap was observed between the cartilage and GelMA hydrogels, suggesting weak adhesion [supplementary material Figs. S2(a) and S2(c); red arrows]. Moreover, cell-free GelMA-GC hydrogel also exhibited robust cartilage-hydrogel integration, while GelMA hydrogel failed [supplementary material Figs. S2(e) and S2(f)]. This provides additional confirmation of the push-out test results, supporting those findings. Although we did not investigate the migration of encapsulated chondrocytes from our hydrogel to the cartilage, a high percentage of viable cells were observed in both hydrogel systems after 28 days of culture [supplementary material Figs. S2(a)–S2(d)]. Based on these data, we hypothesize that GelMA-GC hydrogels will facilitate cell migration to the tissue-construct boundary after *in vivo* implantation, which may enhance the healing process and functional outcomes. Since the hydrogel is biodegradable, it is logical to assume that any mechanical stability provided by this integration will be temporary. However, substantial integration of cell-laden hydrogel into cartilage defects immediately upon application is also required to initiate matrix accumulation at the interface. Since our cell-laden hydrogel demonstrated strong integration with native cartilage *in vitro* even after 28 days of culture, we anticipate that this hydrogel system will enhance the matrix accumulation in the cartilage–hydrogel interface and provide secure integration with native cartilage after *in vivo* application. However, further study is required to validate our hypothesis.

Chondrocytes in GelMA and GelMA-GC hydrogels remained viable (>70%) and homogenously distributed throughout 28 days of *in vitro* culture in free-swelling and loaded conditions [Figs. 2(a) and 2(b), supplementary material Fig. S3(c)]. Chondrocytes in this study retained their rounded morphology, suggesting that their chondrogenic nature was preserved [Fig. 2(a)]. These findings indicate that our hydrogel system is cytocompatible. Lim *et al.* also demonstrated that Ru/SPS (0.2 mM Ru/2 mM SPS) cross-linking systems can preserve high cell viability (>80%) in human articular chondrocytes after 35 days of culture.^51^ GelMA-GC hydrogels cultured in both conditions had comparable DNA content on day 28 compared to GelMA hydrogels [Fig. 2(c)]. DNA content of GelMA-GC hydrogels was considerably lower than GelMA hydrogels on day 1, yet the cell viability and cell density in the live/dead images were similar between groups (Fig. 2). This suggests an underestimate of DNA content in GelMA-GC by the DNA assay at day 1, which is likely due to complexation between the positively charged GC and negatively charged DNA, a phenomenon that is affected by both concentration and pH.^67–69^ DNA content was similar between groups on day 28 [Fig. 2(c)], however, perhaps indicating less free GC to bind with DNA at later time-points. Mechanical loading can induce cell proliferation;^70–72^ although this was not found in our investigation, the overall picture revealed comparable cell viability in all groups following the cessation of the loading period on day 28 of culture [Figs. 2(a) and 2(b)]. Since we only uniaxially loaded the cartilage-hydrogel constructs intermittently for 14 days, it may have been insufficient to promote cellular proliferation.

Chondrocytes use mechano-transduction pathways to sense the mechanical stimuli, regulate gene expression, and control the synthesis and degradation of ECM molecules^15^ while also influencing the integration of implanted material with host cartilage.^18–20^ To determine the response of mechanical stimulation at the cellular level and how it affects the mechanical, adhesive, biochemical, and gene expression of our hydrogel system, we pre-cultured Ru/SPS cross-linked GelMA and GelMA-GC hydrogel-cartilage constructs for 14 days and then uniaxially loaded them for another 14 days in a custom bioreactor.^73^ Due to the similarity of human and bovine articular cartilage in terms of thickness,^74^ and the need for large amounts of cartilage of the same thickness, bovine chondrocytes and articular cartilage were used as the model system for our investigation. We selected the loading regime for this study based on previously published studies. Chondrocyte biosynthesis was found to be stimulated by dynamic stimulation at physiological frequencies of 1 Hz, as discovered by Lee and Bader^75^ and independently validated by other research groups.^26,76–78^ Moreover, it has been found that short intermittent loading (1–5 h/day) improves chondrogenesis,^46,79^ while continuous dynamic stimulation has minimal^80^ or even detrimental effects on chondrogenesis^81^ in most culture systems. According to earlier research, in contrast to confined loading conditions, unconfined mechanical loading resulted in more uniform mechanical signals across the tissue-engineered construct.^82^ However, protective pericellular matrix must be synthesized following encapsulation in hydrogel-based constructs for chondrocytes to respond appropriately to mechanical stimuli.^22^ Previously published data demonstrated that an extended static preculture time of 14 days was required to enhance the expression of chondrogenic marker genes ACAN and COL2A1 in response to loading compared to 7 days of preculture.^22^ Inhibitory effects of loading at early time periods may be related to the absence of a pericellular matrix which may constitute a cellular stress response^83^ rather than facilitating mechano-transduction.^22^ Strain and shear amplitude are the additional characteristics that influence the properties of tissue-engineered cartilage. Knee cartilage is only subjected to 7%–10% compression during walking;^84,85^ however, severe mechanical stresses occur in other activities and are linked to chondrocyte and cartilage pathogenesis.^86^ Earlier research on bovine cartilage explants indicated stimulatory effects of compression levels lower than 5% strain;^87^ however, this lower strain showed non-significant influence on chondrogenic marker gene expression in human chondrocyte-laden alginate hydrogels.^46^ A significant level of chondrogenic marker gene expression was observed at 1.5 mm shear amplitude and 30% compressive strain in GelMA-HAMA hydrogels, yet some evidence of wear and tear were observed. Based on those observations, we used 10% compressive strain stimulation for 14 days at a frequency of 1 Hz for 1 h per day. A static 1 Newton (N) force was applied to all constructs in each loading cycle to confirm the contact of bioreactor pistons with all samples. The maximum force applied to the 24 constructs was consistent over the first 12 days of bioreactor loading (2.3 ± 0.4 N). However, we observed an increasing trend of force over the last 2 days of culture (to 3.5 N on day 13 and 5 N on day 14), which could indicate the early stages of establishment of mechanically functional matrix (supplementary material Table S1).

The extent of *in vitro* maturation of tissue-engineered construct is a significant determinant for successful *in vivo* implantation.^88^ In our investigation, GAG accumulation in all constructs was significantly higher after 28 days, while loaded GelMA hydrogels accumulated significantly more GAG than any other group at 28 days [Fig. 3(a)]. In addition, after short-term loading of the cartilage-hydrogel constructs, GAG content normalized to wet weight increased exclusively in GelMA hydrogels. However, the GAG/wet weight values of free-swelling GelMA, GelMA-GC, and loaded GelMA-GC hydrogels were comparable [Fig. 3(b)]. Furthermore, when retained GAG was normalized to DNA content, both hydrogel groups showed a consistently increasing trend from day 1 to day 28 of culture indicating the presence of more metabolically active cells [Fig. 3(d)]. Since GAGs are highly hydrophilic and rapidly elute in solution from tissue-engineered constructs,^89^ we also quantified the amount of GAG secreted to the culture media to investigate the capacity of the hydrogel matrix to retain GAG. All free-swelling and loaded hydrogels released similar amounts of GAG into the culture medium [Fig. 3(e)]. However, the ratio of secreted to retained GAG was comparable across construct types and culture conditions at day 28, except for loaded GelMA constructs, which retained a more significant amount of GAG [Figs. 3(b) and 3(e)]. This suggests that total GAG production and retention capacities were the highest in the loaded GelMA hydrogels. Contrary to our expectation, mechanical stimulation did not significantly increase GAG release into the medium, demonstrating that the retention ability primarily depends on molecular interactions with the ECM and may not be affected by loading.

The ECM of articular cartilage is composed primarily of collagen II and considerable quantities of proteoglycans such as aggrecan.^90^ Collagen II is a key marker for the development of hyaline-like articular cartilage, which accounts for 90%–95% of the collagens in the articular cartilage matrix.^91^ On the other hand, collagen I is a fibrocartilage marker produced by dedifferentiating articular chondrocytes,^92^ and its expression in tissue-engineered cartilage is undesirable. Therefore, immunofluorescence was utilized to visualize the accumulation of hyaline cartilage markers collagen II and aggrecan, as well as chondrocyte dedifferentiation marker collagen I in our hydrogel system. Both GelMA and GelMA-GC constructs supported the production and accumulation of hyaline cartilage markers, and uniaxial loading further enhanced these markers (Fig. 4, supplementary material Fig. S4). We determined the expression of fibrocartilage associated marker (COL1A2) and chondrogenic marker (COL2A1 and ACAN) genes using qRT-PCR at the end of the culture period. After 28 days of free-swelling culture, the expression of the COL1A2 gene was comparable in both hydrogels. However, the expression of COL2A1 and ACAN genes was considerably lower in free-swelling GelMA-GC hydrogels compared to GelMA hydrogels [Figs. 5(b) and 5(c)]. Similar expressions of COL1A2, COL2A1 and ACAN in free-swelling and loaded GelMA hydrogels suggest no effect of loading on relevant gene expression at 28 days of culture (Fig. 5). While loading also did not affect COL2A1 and ACAN expression in GelMA-GC hydrogels, it significantly lowered COL1A2 expression in this group (Fig. 5). Decrease in the fibrocartilage associated marker COL1A2 in loaded GelMA-GC hydrogels, further suggests the potential of this hydrogel system and the effectiveness of loading for cartilage tissue engineering.

This *in vitro* study demonstrated that intermittent short-term mechanical stimulation enhanced the mechanical and adhesive properties of GelMA-GC hydrogels. Coupled with the knowledge that mechanical stimulation at physiological range contributes to ECM synthesis and remodeling in joints,^93^ it is hypothesized that joint movement following *in vivo* application of GelMA-GC hydrogels would enhance ECM production within the GelMA-GC hydrogel and at the cartilage–hydrogel interface. However, *in vivo* investigations are required to confirm or refute this hypothesis.

## CONCLUSION

Tissue-engineered constructs for cartilage defect repair and regeneration must have adequate mechanical properties and the ability to integrate securely with the native tissue. This study provides proof-of-concept for utilizing an injectable, visible-light cross-linked, mechanically robust, highly adhesive GelMA-glycol chitosan hydrogel. The preliminary *in vitro* study showed the potency of this hydrogel system, creating a secure integration with bovine cartilage in cell-free and cell-laden conditions. In addition, when subjected to short-term mechanical stimulation, this hydrogel system showed promising outcomes necessary for cartilage repair and regeneration. Based on the significant outcomes in terms of mechanical stability, adhesion, cytocompatibility, and matrix accumulation, this hydrogel may be suitable as a future alternative to existing clinical bioadhesives used in cartilage repair. Nonetheless, additional *in vitro* and *in vivo* studies are necessary to demonstrate the long-term efficacy of this hydrogel system for cartilage repair and regeneration.

## METHODS

### Articular cartilage ring preparation

**FIG. 7.**
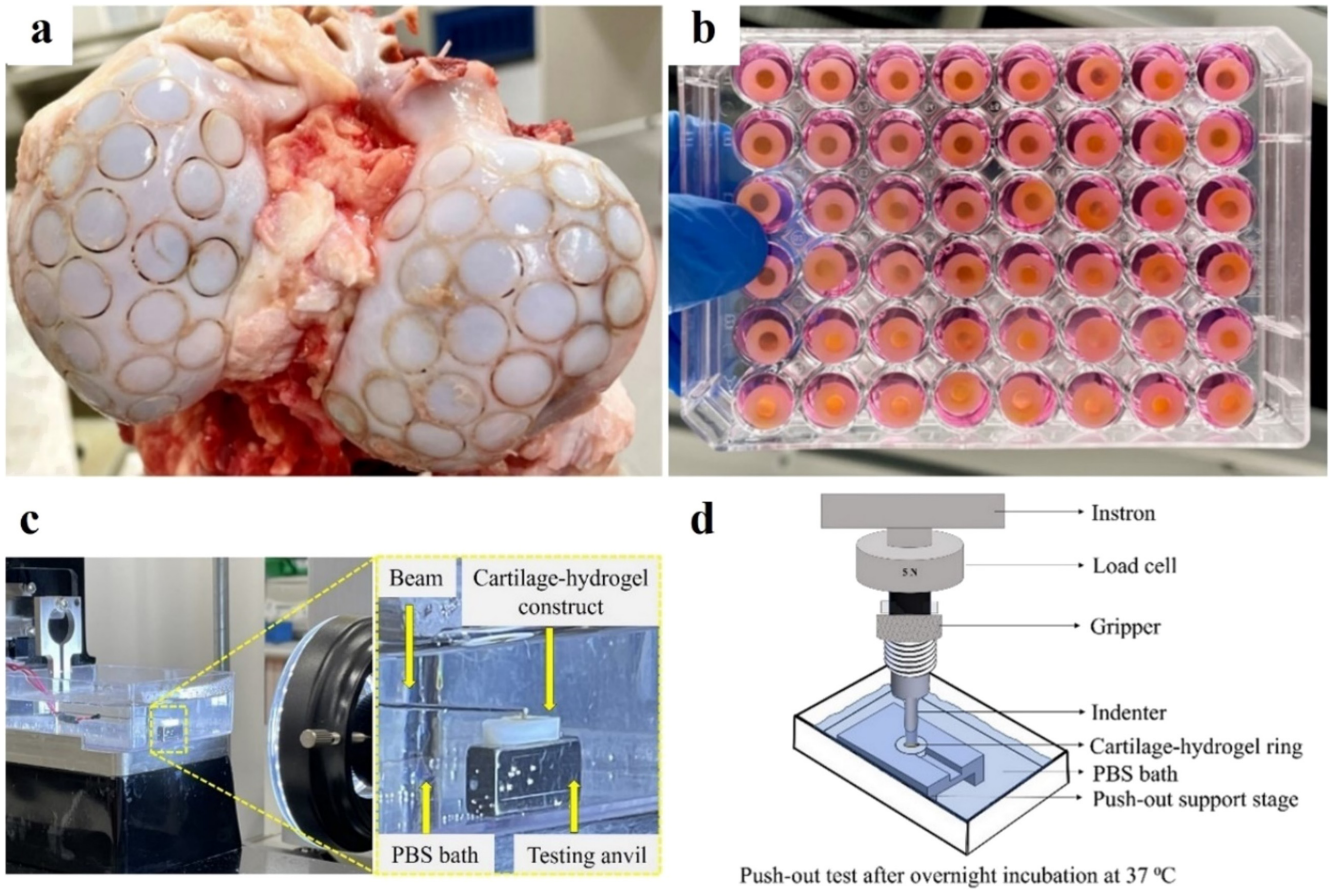
Cartilage-hydrogel constructs preparation, mechanical and adhesive tests. (a) Preparation of osteochondral constructs from bovine stifle joints by trephining. (b) Culture of cartilage-hydrogel constructs in 48-well plates with chondrogenic media. (c) Micro-indentation test performed in a cell-scale micro-tester at 37 °C in PBS. (d) Push-out test performed in Instron 5567 with a 5 N load cell at 37 °C in PBS.

Articular cartilage rings were prepared from 1- to 3-year-old bovine stifle joints acquired fresh from a butcher. Joints were kept moist during transport and extraction by rinsing them with sterile phosphate buffered saline (PBS; pH 7.4) (Thermofisher Scientific, Australia) containing penicillin (100 U/mL) and streptomycin (100 *μ*g/mL) (all from Gibco™, Thermofisher Scientific, Australia). The osteochondral constructs (height: 10–15 mm, diameter: 9 mm) were extracted from the femoral condyles and the trochlear groove by trephining [Fig. 7(a)] and transferred into a custom-made mold to remove the superficial layer with a sterile scalpel. The osteochondral constructs were then sliced into cartilage disks (thickness: ∼1.6 mm) using a custom mold and sterile scalpel [supplementary material Figs. S5(a)–S5(c)]. All cartilage disks were prepared aseptically and stored at −20 °C. The disks were thawed for 10 min at 37 °C in PBS containing penicillin (100 U/mL) and streptomycin (100 *μ*g/ml) before use, and a central defect (diameter: 4 mm) was created using a biopsy punch (Kai Medical, Japan) guided by a custom-made mold [supplementary material Figs. S5(c)–S5(e)]. The cartilage rings were stored at room temperature (thickness: ∼1.6 mm, outer diameter: 9 mm, and inner diameter: 4 mm) in sterile PBS with penicillin (100 U/ml) and streptomycin (100 *μ*g/ml) until use.

### Bovine articular chondrocyte isolation and expansion

Chondrocytes were isolated from fresh, full-thickness bovine articular cartilage (1–3 years of age) of the lateral and medial femoral condyles, as reported elsewhere.^22,94^ Chondrocytes were expanded in Dulbecco's Modified Eagle Medium (DMEM) (Gibco™, Thermofisher Scientific, Australia) supplemented with 2 mM GlutaMAX™, 10 mM 4–(2-hydroxyethyl)-1-piperazineethanesulfonic acid (HEPES), 0.1 mM MEM non-essential amino acid solution, 50 *μ*g/ml penicillin/streptomycin, 0.25 *μ*g/ml amphotericin B (Fungizone^®^) (all from Thermofisher Scientific, Australia), 0.4 mM l-proline, 0.1 mM l-ascorbic acid (both Sigma-Aldrich, Australia), and 10% fetal bovine serum (FBS) (Hyclone, USA).

### Hydrogel preparation

Sterile freeze-dried GelMA polymer (gelatin methacryloyl, porcine skin, type A, 80% Degree of Functionalization, Gelomics Pty Ltd., Australia) was dissolved in autoclaved PBS at 35% (w/v) for 24 h at 37 °C in a hybridization oven (HO35, Ratek, Australia) with constant rotation. Glycol chitosan (GC) (≥60% titration, crystalline, Sigma-Aldrich, USA) was dissolved in PBS to obtain a 3% (w/v) stock solution and then filter-sterilized (Filtropur S 0.22, Sarstedt, Germany). Stock solutions of photo-initiators (50 mM Ru (tris (2,2′-bipyridyl) dichlororuthenium (II) hexahydrate ([RuII(bpy)_3_]^2+^) and 1 M SPS (sodium persulfate) (all Sigma-Aldrich, USA) were prepared in PBS and filter-sterilized (Filtropur S 0.22, Sarstedt, Germany) on the day of hydrogel construct preparation. Prior to cross-linking, hydrogel precursor solutions of 15% w/v GelMA, and 15% w/v GelMA with 1% w/v GC (GelMA-GC), were prepared in PBS with 0.5 mM Ru/20 mM SPS.

### Cartilage-hydrogel construct preparation

Bovine articular chondrocytes (passage 1, seeding density of 8.3 × 10^6^ cells per mL of hydrogel) were encapsulated in GelMA and GelMA-GC hydrogel precursor solutions prepared as outlined in the section of hydrogel preparation. The cell-laden hydrogel precursor solution was injected into the central defect (diameter: 4 mm) of cartilage rings and the leakage or overflow was prevented by placing the rings in a polydimethylsiloxane (PDMS) sheet with barriers. Hydrogel cross-linking was performed in an LED cross-linker (Luna Crosslinker, Gelomics, Australia) for 5 min at 405 nm with an intensity of 9 mW/cm^2^. Cell-free cartilage-hydrogel constructs were also prepared using the same procedure. The samples were cultured in media containing serum-free high-D-glucose basal chondrocyte medium supplemented with bovine serum albumin (BSA; 1.25 mg/ml), Insulin–Transferrin–Selenium (ITS-G; 100 × dilution), dexamethasone (0.1 *μ*M) (all Sigma-Aldrich, USA) and transforming growth factor beta 3 (TGF-β3; 10 ng/ml) (GroPep, Australia) for 28 days at 37^ ^°C. The media was changed twice a week, and samples were cultured with and without mechanical stimulation.

### Intermittent uniaxial mechanical stimulation of cartilage-hydrogel constructs

Cell-laden hydrogel-cartilage constructs were either cultured under free swelling conditions for 28 days, or pre-cultured for 14 days, followed by an intermittent uniaxial loading daily for 1 h at 1 Hz frequency with a compressive strain of 10% of the construct height in a custom-made dynamic mechanical stimulation bioreactor^73^ for another 14 days. In the bioreactor, printed medical-grade resin (BioMed Clear, Formlabs, USA) rings (thickness: 1 mm, outer diameter: 15.2 mm, and inner diameter: 10 mm) were placed at the bottom of each well of 24-well plates to loosely confine the cartilage-hydrogel constructs and prevent slippage during uniaxial loading [supplementary material Fig. S5(f)]. Throughout dynamic culture, bioreactors were kept at 37 °C in a sterile, humidified atmosphere of 5% CO_2_, with medium changes twice a week. Conditioned medium was collected 1 h after the end of the loading cycle and stored at –20 °C before quantification of GAG with the DMMB assay described in the biochemical analysis section. A static 1 N force was applied to all 24 constructs in each loading cycle to ensure contact of the bioreactor pistons with the sample surface. The vertical actuator position and force vs time data (initial and end parts of each cycle) were recorded to observe the maximum force applied on 24 constructs throughout the couture period (supplementary material Table S1). Two hours after the end of the last loading cycle (day 14 of loading), constructs were terminated for mechanical tests (micro-indentation), adhesion test (push-out test), cell viability, biochemical analysis, immunohistochemistry, and gene expression analysis detailed in the subsequent sections. Samples used for micro-indentation tests were utilized in the adhesion test, cell viability test, and biochemical analysis, whereas samples for immunofluorescence and gene expression analysis were collected separately.

### Mechanical test: Micro-indentation

The mechanical characteristics of cell-free and cell-laden hydrogel constructs were evaluated by micro-indentation compression testing on days 1 and 28 of free swelling and 14 of preculture and day 14 loading (day 28 of overall cell culture), as described elsewhere.^95^ Micro-indentation was performed at a constant jogging speed (4 *μ*m/s) using a high-precision piezoelectric actuator-controlled micro-compression system (CellScale Microtester, Canada), at 37 °C in a water bath containing PBS supplemented with penicillin (100 U/ml) and streptomycin (100 *μ*g/ml). Briefly, each cartilage-hydrogel construct was placed on a testing anvil, and the microbeam was positioned just above the center of the hydrogel sample [Fig. 7(c)]. The hydrogels were indented with a 0.5 mm Zirconium oxide bead (ZROB05, Next Advance, USA), which was attached to the end of a 0.5588 mm diameter cantilevered steel microbeam [Fig. 7(c)]. The microbeam generated cyclic compression cycles by applying a vertical force with amplitudes of 10% of the sample height. Each cycle consisted of a 30-second “load” phase followed by a 30-s “recovery” phase, with no rest in between. Three cycles were performed for each hydrogel sample (n = 4). During the experiment, the indentation force (F) and indentation depth (δ) were continually estimated based on the optically recorded deflection of the cantilevered beam's indenter end and the piezo-controlled z-displacement of the cantilevered beam's fixed end. We monitored the change in hydrogel thickness during compression and measured the jogging force required to generate a force-displacement curve. Using the Hertz contact model^43^ for a spherical indenter, experimental force-indentation depth data were fitted to determine the elastic modulus (E) of each sample, by the following equation:

$$E=\frac{3\left(1-v^{2}\right)F}{4R^{0\cdot 5}\delta^{1\cdot 5}},$$ (1)

where R is the radius of the indenter and ν is the Poisson's ratio (assumed to be 0.44^96^). Samples were utilized for push-out test immediately after mechanical loading.

### Adhesion test: Push-out

The adhesive strength of GelMA and GelMA-GC hydrogels to the articular cartilage was evaluated by conducting a push-out test in free-swelled (day 1 and 28), and bioreactor-loaded (day 14) samples based on optimized previously published protocol.^44,45^ The test was performed at 37 °C with an Instron 5567 (Instron 5567, USA) electromechanical test device equipped with a 5 N load cell at a speed of 0.01 mm/s. In the push-out test, a 3D-printed indenter (printed with Biomed Clear on a Form 3B+ printer, FormLabs, USA) with a 3.5 mm diameter was used to dislodge the cross-linked hydrogel samples from the cartilage rings [Fig. 7(d), supplementary material Fig. S6(a)]. Samples were held securely in place using a custom-made support stage [Fig. 7(d), supplementary material Fig. S6(b)] submerged in an immersion bath containing PBS supplemented with penicillin (100 U/ml) and streptomycin (100 *μ*g/ml) at 37 °C. The rim of the upper plate hole confined the cartilage ring during testing. The contact area between the hydrogel and the inner surface of the cartilage defect was calculated as the cartilage defect's circumference multiplied by the hydrogel's height. The push-out strength was determined by normalizing the maximum force to remove the hydrogel sample from the cartilage ring to the contact area. Four cartilage rings (n = 4) were used for each group and condition. Following the test, hydrogel samples were collected and kept sterile in PBS containing (100 U/ml) and streptomycin (100 *μ*g/ml) for cell viability and biochemical analysis.

### Cell viability

The viability of chondrocytes in GelMA and GelMA-GC hydrogels was determined on days 1 and 28 of free-swelling and day 14 of intermittent uniaxial loading (total 28 days of culture) using a live/dead assay described elsewhere.^97^ Briefly, after the push-out test, hydrogel constructs were sliced into two by a sterile scalpel, washed with PBS, and then incubated for 3 min at room temperature in PBS solution containing fluorescein diacetate (FDA; 1 *μ*g/ml) and propidium iodide (PI; 1 *μ*g/ml) (both from Sigma-Aldrich, USA). The samples were rewashed with PBS, and four Z-stack images per sample were acquired using a Zeiss Axio microscope (Carl Zeiss Axio Imager M2, GmbH, Germany) at each time point for each group (n = 4) and analyzed using Image J software (National Instruments, USA). Cell viability reported as a percentage of the total number of cells that were alive. Whole-slice images of hydrogels were also captured to represent an overall view of the impact of uniaxial loading on the top and bottom parts of hydrogel in cartilage-hydrogel constructs. To comprehensively examine the integration and cell viability of distinct cartilage-hydrogel areas, whole cartilage-hydrogel constructs were imaged on day 28 of culture with a Zeiss Axio microscope following a live/dead assay.

### Biochemical analysis

GelMA and GelMA-GC hydrogel constructs were weighed and digested overnight at 56 °C in phosphate-buffered EDTA (PBE; pH 7.1) containing 0.5 mg/ml Proteinase K (Thermofisher Scientific, Australia) in a thermo-shaker (Eppendorf AG 22331 Hamburg, Germany) for the biochemical measurement of glycosaminoglycans (GAG) and DNA content. The amount of GAG in the digested samples was determined using a dimethylmethylene blue (DMMB) assay.^98^ The absorbances of hydrogel digest were measured at 525 and 595 nm using a CLARIOstar microplate reader (BMG Labtech, Australia) and compared to a chondroitin sulfate (Sigma-Aldrich, Australia) standard curve at concentrations ranging from 0 to 100 *μ*g/ml prepared in PBE. At each media change, GAG secreted to the culture medium was also quantified. The GAG content of media was measured using the DMMB (pH 3) assay utilizing chondroitin sulfate standard curve at concentrations ranging from 0 to 100 *μ*g/ml prepared in media. The DNA content of digested hydrogel samples was measured using the Quant-iT PicoGreen^®^ dsDNA quantification assay (Life Technologies, USA) according to the manufacturer's instructions.

The biosynthetic activity of the cells was assessed by normalizing the GAG quantification to the double-stranded DNA (dsDNA) content determined by a Quant-iT PicoGreen reagent (Life Technologies, USA) according to the product protocol. Standard curves were prepared at concentrations ranging from 0 to 1 *μ*g/ml in 1× Tris-EDTA (TE) buffer (Sigma-Aldrich, Australia). The dsDNA content was quantified by obtaining the fluorescence intensities at an excitation wavelength of 485/20 nm and an emission wavelength of 528/20 nm using a CLARIOstar microplate reader.

Even with comparable cell viability to GelMA-only hydrogels, we detected a decrease in the DNA content in GelMA-GC hydrogels compared to their respective controls in an experiment suggesting a possible interaction between GC and DNA^99^ standard or the fluorescent dye. To account for this interaction, we calibrated the normal DNA standard with the GelMA-GC-containing DNA standard to calculate the multiplying factor to determine the DNA content of GelMA-GC hydrogels. First, cell free GelMA-GC hydrogels were weighed and digested overnight at 56 °C in phosphate-buffered EDTA (PBE; pH 7.1) containing 0.5 mg/ml Proteinase K in a thermo-shaker. DNA standards were prepared at concentrations ranging from 0 to 1 *μ*g/ml in 1X Tris-EDTA (TE) buffer, and GelMA-GC-DNA standards were prepared by adding 5 *μ*l of GelMA-GC hydrogel digest with 95 *μ*l of each DNA standard. Standard curves were generated using the Quant-iT PicoGreen^®^ dsDNA quantification assay according to the manufacturer's instructions. Finally, the multiplying factor for determining the DNA content of GC-containing hydrogels was determined through the calibration of a DNA standard with the GelMA-GC-containing DNA standard.

### Immunofluorescence analysis

Samples for immunofluorescence imaging were prepared according to a previously established methodology^100^ after appropriate adjustment. Following culture on days 1 and 28 of free-swelling and day 14 of uniaxial loading (total 28 days culture), cartilage-hydrogel constructs were fixed with 4% (w/v) paraformaldehyde (PFA) (Sigma-Aldrich, Australia) in PBS at room temperature for 1 h. Cartilage-hydrogel constructs were immersed in increasing concentrations of sucrose (Sigma-Aldrich, Australia) solution [10% (w/v) for 1 h, 15% (w/v) for 1 h, 20% (w/v) overnight, 30% (w/v) for 8 h, and 30% (w/v) + Optimal Cutting Temperature compound (OCT; Sakura, Finetek, Japan) overnight at 50:50 ratio] at 4 °C. Following this, the constructs were immersed in OCT only compound for 8 h at room temperature, frozen with liquid nitrogen (−196 °C), and kept at −20 °C until use. Cryosections of constructs with a thickness of 10 *μ*m were generated in poly-l-lysine coated slides (Super-Frost Plus Slides, Thermo Scientific™, USA) using a cryostat (Cryostar NX70, Thermofisher Scientific, Australia) and stored at −20 °C after overnight drying at room temperature. On the day of immunostaining, the cryo-sectioned samples were thawed by wrapping in a towel for a few minutes, and the hydrophobic borders were drawn using a DAKO pen (Ngaio Diagnostics, New Zealand). The OCT was removed from the cryosection slides by incubating them for 5 min in PBS at room temperature. Cryosections for staining collagens I, II, and aggrecan were treated with 0.1% (w/v) hyaluronidase (Sigma-Aldrich, Australia) in PBS at 37 °C for 30 min to retrieve antigens, followed by two 4-min rinses with 0.1% (v/v) Tween 20 (Sigma-Aldrich, France) in PBS and one 4-min rinse in PBS. Primary antibodies for Collagen I (Monoclonal mouse collagen type I; DSHB Hybridoma Product 8–3A5; deposited to the DSHB by De Blas, Angel L., USA) final concentration 3 *μ*g/ml in PBS with 2% (v/v) goat serum (Gibco™, Thermo Scientific™, USA), Collagen II (monoclonal mouse collagen type II; DSHB Hybridoma Product II-II6B3; deposited to the DSHB by Linsenmayer, T.F., USA) final concentration 5 *μ*g/ml in PBS with 2% (v/v) goat serum), and Aggrecan (monoclonal mouse aggrecan core protein; DSHB Hybridoma Product 12/21/1-C-6; deposited to the DSHB by Caterson, B., USA); final concentration 5 *μ*g/ml in PBS with 2% (v/v) goat serum were administered overnight in a humidified chamber at 4 °C. Sections rinsed twice with 0.1% (v/v) Tween 20-PBS wash buffer and once with only PBS for 4 min each wash. Secondary antibody [Goat anti-Mouse IgG (H + L) Cross-Adsorbed Secondary Antibody, Alexa Fluor™ 488, Thermo Scientific™, USA] for Collagen I, II and aggrecan [final concentration 4 *μ*g/ml in PBS with 2% (v/v) goat serum], as well as DAPI (final concentration 5 *μ*g/ml in PBS) (Thermo Scientific™, USA), were administered for 1 h at 4 °C in the dark. Sections were rinsed twice with 0.1% (v/v) Tween 20-PBS wash buffer and once with only PBS for 4 min each time at the end of secondary antibody and DAPI application. Slides were air dried and coversliped with ProLong Gold (ProLong™ Gold Antifade Mountant, Thermo Scientific™, USA), left for curing overnight in the dark at room temperature and then stored at room temperature. The samples were photographed using a ZEISS Axio Observer 7 microscope (Carl Zeiss Microscopy, GmbH, Germany). The absence of nonspecific staining was verified by using sections treated with secondary antibodies as negative controls.

Immunofluorescence images were processed using Image J software. First, the image taken by a ZEISS Axio Observer 7 microscope was opened in the Image J software, and the channels were split. The Hyper-Stack command was applied to each channel, followed by the stack to Z-project command. The brightness of each channel was adjusted within a specified range, and the image was saved in RGB format. Following this, the “Subtract Background” command was employed with a rolling ball pixel radius of 10. All commands listed were applied to each channel image, and the final image was processed by adjusting brightness and contrast using Adobe Photoshop (Adobe Photoshop CC 2017). The processed images were then used to generate the representative figures.

### Gene expression analysis

To evaluate temporal variations of gene expression following compression at 10% strain of construct height, GelMA and GelMA-GC hydrogels were sampled for total RNA isolation 2 h after loading, since prior study showed the highest mRNA peak occurred 2 h post-loading. Cell-laden hydrogel constructs were pushed out from the cartilage rings by the indenter [diameter: 3.5 mm, supplementary material Fig. S2(a)] used in the push-out test and transferred to 1 ml TRIzol reagent (ThermoFisher Scientific^TM^, USA), snap-frozen in liquid nitrogen, and stored at −80^ ^°C until the analysis.

Total RNA was extracted according to our previously reported method.^101^ Briefly, the hydrogels were homogenized in 1 ml TRIzol reagent by repeatedly pressing for 15–20 times them through a 19-gauge needle attached to a 1 ml syringe. Super-Script™ IV VILO™ (ThermoFisher Scientific^TM^, USA) was used to synthesize complementary DNA (cDNA) from the total RNA recovered. SybrGreen^®^ Mastermix (ThermoFisher Scientific^TM^, USA) and a QuantStudioTM 7 Flex Real-Time PCR machine (Applied Biosystems, ThermoFisher Scientific^TM^, USA) were used for quantitative reverse transcription real-time polymerase chain reaction (qRT-PCR). The cycle threshold (Ct) value of target gene was normalized using the comparative Ct method (2^−ΔCt^) relative to the geometric mean of the expression of the housekeeping genes RPL13A and B2M. The sequences of the forward (F) and reverse (R) primers (5′ - 3′) utilized in qRT-PCR are listed in supplementary material Table S2.

### Statistical analysis

All data were analyzed using GraphPad Prism (version 9; GraphPad, USA). Where appropriate, one-way or two-way ANOVA with Tukey's honest significant difference post-hoc test was employed to compare group means. Differences were considered significant when *p* < 0.05 for all tests and are indicated in figures using Roman numerals.

## SUPPLEMENTARY MATERIAL

See the supplementary material for the following details: Cross-sectional view of cell-laden free-swelling and loaded GelMA and GelMA-GC hydrogels cross-linked by Ru/SPS photo-initiators at 405 nm on day 28 (Fig. S1); cartilage-hydrogel integration of cell-laden and cell free, free-swelled and loaded GelMA and GelMA-GC hydrogels cross-linked by Ru/SPS photo-initiators at 405 nm on day 28 (Fig. S2); biochemical properties of cell-free, cell-laden, free-swelled, and loaded GelMA (15%; w/v) and GelMA-GC (GelMA 15% and GC 1%, w/v) hydrogels cross-linked with Ru/SPS photo-initiators at 405 nm (Fig. S3); extracellular matrix production in free-swelling cultured constructs at day 1 (Fig. S4); technical drawing and dimensions for the custom-made plastic mold for articular cartilage slice and rings preparation (Fig. S5); technical drawing and dimensions of custom-made indenter and support stage push-out test (Fig. S6); bioreactor loading data of culture plate 1 and plate 2 (supplementary material Table S1); forward (F) and reverse (R) primer sequences for qRT-PCR (supplementary material Table S2).

## Data Availability

The data that support the findings of this study are available from the corresponding authors upon reasonable request.
